# Advancing vitiligo care in Russia: landscape, lessons, and a scalable digital health strategy

**DOI:** 10.3389/fmed.2025.1656972

**Published:** 2025-08-26

**Authors:** Maria Borodina, Julia Sigova, Konstantin Lomonosov, Ekaterina Suvorova

**Affiliations:** ^1^Russian Children's Clinical Hospital, Pirogov Russian National Research Medical University, Moscow, Russia; ^2^Department of Neonatology, Faculty of Continued Medical Education, Pirogov Russian National Research Medical University, Moscow, Russia; ^3^Department of Dermatology and Communicable Diseases, Ministry of Health, I.M. Sechenov First Moscow State Medical University, Moscow, Russia; ^4^Peoples' Friendship University of Russia named after Patrice Lumumba, Moscow, Russia

**Keywords:** vitiligo, teledermatology, Russia, public health, artificial intelligence (AI)

## 1 Introduction

Based on extrapolated prevalence estimates, vitiligo likely affects between 730,000 and 1.46 million people in Russia, yet care remains fragmented and inconsistently available, particularly outside major urban centers. Although some locally developed therapies, such as intramuscular acridone acetic acid (Na-AAA) (1), have shown efficacy in halting disease progression, they remain largely unknown beyond national borders. Building on the foundation laid in “Vitiligo: A Call for Paradigm Shift Toward Comprehensive Patient Care,” (2) this Opinion highlights gaps in Russia's vitiligo care landscape and presents a pragmatic, AI-enabled teledermatology model designed to deliver equitable care across the country's 11 time zones. Importantly, the proposed model may also serve as a blueprint for other regions with similarly vast, sparsely populated territories and limited access to dermatological expertise.

## 2 Epidemiology and burden in Russia

A 2024 global modeling analysis that included Russian data sources estimated the self-reported lifetime prevalence of vitiligo in Central Europe—a region demographically comparable to Russia—at 0.55% (95% credible interval: 0.33–0.92%) (3). Other multinational population-based surveys in Europe report adult prevalence between 0.6% and 1%, particularly when undiagnosed cases are included alongside diagnosed ones (4, 5). Extrapolating these findings, a reasonable prevalence range for adults in Russia is 0.5–1% of the population. Applied to Russia's 2025 population of 146,028,325, this corresponds to ~730,000–1,460,000 individuals living with vitiligo.

Regional variations may exist: for example, the Ministry of Health of Tatarstan has reported a prevalence of around 2% among its residents (6). A nationwide study to measure vitiligo prevalence in Russia is currently underway, with results pending (7). Surveys conducted in Moscow, St Petersburg, and Kazan show that more than 70% of respondents experience moderate or severe quality-of-life impairment (DLQI ≥ 6) (8, 9). Common stressors include workplace discrimination, difficulty establishing relationships, and reluctance to seek treatment because vitiligo is often perceived as “cosmetic” rather than medical.

## 3 Current management landscape

Vitiligo management in Russia is shaped by geographic, socioeconomic, and regulatory factors. The most common Fitzpatrick skin types are I and II in the west and north, and type III in the south and east—reflecting the country's ethnic and geographic diversity.

### 3.1 Clinical guidelines and therapeutic algorithm

The Federal Clinical Guidelines on Vitiligo (10) remain the primary reference for clinicians and recommend a tiered approach:

#### 3.1.1 Topical glucocorticoids (Level A)

First-line for limited ( ≤ 10% BSA) and segmental vitiligo. Agents include methylprednisolone aceponate, clobetasol propionate, and betamethasone dipropionate.

Adults: high to very-high potency (up to 2–3 months); children: moderate to high potency. Intermittent regimens (e.g., 2 weeks on, 2 weeks off) help reduce side effects.

#### 3.1.2 Topical calcineurin inhibitors (Level A–C)

Used when steroids are ineffective or contraindicated. Tacrolimus 0.03% or pimecrolimus 1% applied twice daily for ≥3 months. Widely used off-label due to low risk of skin atrophy.

#### 3.1.3 NB-UVB phototherapy (311 nm, Level A)

Initiate at 100–250 mJ/cm^2^, increase by 5–20% per session, 2–3 times weekly. Cumulative doses capped around 35 J/cm^2^ per cycle. Clinical studies report ~45% repigmentation after 6 months.

#### 3.1.4 Systemic corticosteroids (Level C)

Dexamethasone mini-pulse: 0.1 mg/kg on two consecutive days per week for 3 months, followed by tapering—indicated for rapidly progressive disease.

#### 3.1.5 Supportive care

Patients are advised to avoid trauma, sunburn, and emotional stress. Daily broad-spectrum sunscreen (SPF 30–50) is recommended, especially alongside phototherapy to minimize risk of UVB overdosing.

### 3.2 Access to phototherapy

NB-UVB booths are widely available in federal dermatology centers in major cities (e.g., Moscow, St. Petersburg, Novosibirsk), but access in rural areas remains limited. Outpatient phototherapy procedures are not included in the mandatory medical insurance program (OMS). Some patients receive this treatment in public healthcare institutions, while others turn to private clinics. The average cost of one session is ~1,000 RUB (≈$12.50 at time of writing).

However, treatment adherence remains a challenge. Recent clinical observations indicate that only about 68% of patients complete the full course of NB-UVB therapy, with the average number of treatments being 49 sessions (11).

### 3.3 Immunomodulatory therapy: acridone acetic acid (Na-AAA)

Na-AAA, known commercially as Neovir^®^, is a Russian-developed immunomodulatory compound introduced in the 1990s. Synthesized and tested domestically, Na-AAA has been widely used in Russia and several neighboring countries for the treatment of viral infections, autoimmune disorders, and, more recently, as a potential anticancer agent (12). Its integration into Russian dermatological practice reflects a broader tradition of using locally developed small molecules in areas with limited access to Western biologics (13).

A 2014 open-label pilot study found that 10 intramuscular injections (2 mL of Na-AAA 12.5% w/v solution, mixed 1:1 with novocaine) given every 48 h stabilized non-segmental vitiligo in 73% of patients for over a year in clinical study, and over 5 years in follow-up observations. Positive predictors included early-stage disease and limited BSA involvement. Despite its safety, affordability, and decades of clinical use, Neovir^®^ has not been incorporated into international vitiligo treatment guidelines, most likely because large, multicenter trials outside Russia have not been conducted and international regulatory approval has not been actively pursued.

### 3.4 Adjunctive care

- Psychological counseling and cognitive–behavioral therapy are offered *ad hoc* in multidisciplinary private clinics, but are not formally integrated into care pathways (9).

- Camouflage cosmetics and micropigmentation are available commercially but not reimbursed.

- Screening for vitamin D deficiency, autoimmune thyroid disease, and metabolic syndrome is inconsistently performed, despite guideline recommendations.

## 4 Lack of organized patient support

Despite a population of ~147 million, Russia has no national vitiligo patient association. By comparison, the United States (population ~336 million) hosts at least four well-established support organizations—VITFriends, the Global Vitiligo Foundation, Vitiligo Support International, and Vitiligo Bond, among smaller local groups. Local social-media communities provide some support, but geographic fragmentation limits opportunities for sustained peer-to-peer mentorship. Nevertheless, Russian activists and dermatologists celebrate World Vitiligo Day every 25 June, holding public events and media appearances around the country. These grassroots efforts demonstrate appetite for a cohesive national network of vitiligo support groups.

## 5 Interdisciplinary care model: a pragmatic, tele-enabled pathway

Russia's healthcare system is characterized by pragmatic and results-oriented approaches. Across 11 time zones—from Kaliningrad to Kamchatka—building brick-and-mortar dermatology centers in every region is neither feasible nor fiscally sound. Authors hypothesize that a lean, hub-and-spoke model reinforced by teledermatology offers the most practical route to equitable care in vitiligo under such conditions.

### 5.1 Hub-and-spoke, supercharged by teledermatology

- Hubs (≈25–30 state dermatology dispensaries in regional capitals such as Moscow, St Petersburg, Novosibirsk, Yekaterinburg, Krasnoyarsk, Vladivostok) deliver advanced services: NB-UVB, dermatosurgery, and psychosocial counseling.

- Spokes include rural Feldsher-midwife stations (FAPs) and district polyclinics. Front-line clinicians capture standardized smartphone images, upload via secure cloud, and receive a specialist plan within 72 h, regardless of geographic distance.

- Digital backbone: domestic telehealth platforms (e.g., SberHealth, Doktis) leverage Russia's deep pool of IT engineers. AI-triage filters non-urgent cases; dermatologists focus on decision-making, not data entry.

### 5.2 Minimum viable care package

At spoke level:

– Photography protocol: three views per lesion, ruler for scale.

– Basic labs: CBC, TSH, fasting glucose, serum vitamin D.

– First-line therapy: tacrolimus 0.1 % or clobetasol 0.05 % dispensed on site.

– Second-line therapy: add portable NB-UVB devices for home use after in-office training and under distance supervision by a dermatologist and/or during periodic in-person visits.

At hub level:

– NB-UVB protocol: initial 200–300 mJ/cm^2^, +10% per session until a soft holding dose at 2,000–4,000 mJ/cm^2^ for the body and 1,500 mJ/cm^2^ for the face is reached, depending on skin response and erythema,— thrice weekly for the first 3 months, twice weekly thereafter.

– Neovir^®^: 10 injections of 2 mL Na-AAA 12.5% w/v solution (mixed 1:1 v/v with 2 mL of novocaine) every 48 h to arrest rapid progression, if necessary, prior to NB-UVB therapy.

– Quarterly psycho-education webinars streamed nationwide; recordings archived for asynchronous viewing.

### 5.3 Data, quality, and funding

All patient encounters populate a vitiligo module within the state health information system, enabling real-time monitoring of treatment outcomes and resource allocation across regions.

Dermatology consultations in public healthcare institutions are covered under the Mandatory Health Insurance scheme (OMS). In private clinics, however, the cost of a dermatology visit ranges from 2,500 to 7,000 RUB (≈$30–80), depending on the region and the physician's level of expertise.

## 6 AI integration opportunities

AI-based tools hold considerable promise for vitiligo care in Russia, though most remain unvalidated—particularly for the lighter Fitzpatrick skin types (I–III) that predominate across the country. One example is ScanDerm, an AI-assisted platform initially focused on cosmetic and skincare applications but now gradually exploring broader dermatological use cases (14). In 2023, Sechenov 1st Medical University and ScanDerm jointly launched an online service leveraging machine learning to analyze photographs of the skin, hair, and nails. It is designed to alert users to potential dermatologic abnormalities and direct them toward telemedicine or in-person evaluations. Though not vitiligo-specific and lacking formal clinical validation for such use, its image segmentation and texture analysis architecture could contribute to quantifying lesion involvement and tracking disease progression. Preliminary pilot data indicate a reduction of ~25% in unnecessary in-person dermatology visits among stable cases. Integrated within secure telehealth platforms (like SberHealth), such tools could potentially facilitate triage workflows in underserved regions.

Beyond staging and triage, future machine-learning models trained on local genomic and imaging datasets may help identify clinical or molecular predictors of treatment response, laying the groundwork for personalized therapy. Robust clinical calibration across diverse pigmentation profiles will be essential to ensure both diagnostic accuracy and equitable utility, before such tools can be widely adopted in routine vitiligo care.

## 7 Recommendations

- Establish a national vitiligo patient alliance under the Russian Society of Dermatovenereologists and Cosmetologists or other professional organizations.

- Update federal guidelines to include standardized psychosocial screening.

- Expand NB-UVB infrastructure through public–private partnerships in regions with >1 million population.

- Launch a government-funded national vitiligo registry with integrated AI capacity for image analytics and diagnostic assistance.

- Support domestic trials of Na-AAA and related immunomodulators to better define efficacy and indications.

## 8 Conclusion

Russia benefits from established academic dermatology traditions, the availability of underutilized therapies, and an emerging ecosystem of AI developers. Integrating these strengths within a structured interdisciplinary model—and empowering patients through a unified support network—can close current care gaps and improve long-term outcomes for people living with vitiligo. These insights may not only guide improvements in Russia but also inform strategies in other countries facing similar geographic and healthcare challenges.
